# Advanced Consumer Behaviour Analysis: Integrating Eye Tracking, Machine Learning, and Facial Recognition

**DOI:** 10.3390/jemr19010009

**Published:** 2026-01-19

**Authors:** José Augusto Rodrigues, António Vieira de Castro, Martín Llamas-Nistal

**Affiliations:** 1 atlanTTic Research Center for Telecommunication Technologies, University of Vigo, 36310 Vigo, Spain; martin@det.uvigo.es; 2 SIIS-ISEP, Rua Dr. António Bernardino de Almeida 431, 4249-015 Porto, Portugal; avc@isep.ipp.pt

**Keywords:** eye tracking, computer vision, OpenCV, machine learning, facial recognition, consumer behaviour, consumer interest, behavioural data, demographic profiling, market segmentation, marketing strategies, real time insights

## Abstract

This study presents DeepVisionAnalytics, an integrated framework that combines eye tracking, OpenCV-based computer vision (CV), and machine learning (ML) to support objective analysis of consumer behaviour in visually driven tasks. Unlike conventional self-reported surveys, which are prone to cognitive bias, recall errors, and social desirability effects, the proposed approach relies on direct behavioural measurements of visual attention. The system captures gaze distribution and fixation dynamics during interaction with products or interfaces. It uses AOI-level eye tracking metrics as the sole behavioural signal to infer candidate choice under constrained experimental conditions. In parallel, OpenCV and ML perform facial analysis to estimate demographic attributes (age, gender, and ethnicity). These attributes are collected independently and linked post hoc to gaze-derived outcomes. Demographics are not used as predictive features for choice inference. Instead, they are used as contextual metadata to support stratified, segment-level interpretation. Empirical results show that gaze-based inference closely reproduces observed choice distributions in short-horizon, visually driven tasks. Demographic estimates enable meaningful post hoc segmentation without affecting the decision mechanism. Together, these results show that multimodal integration can move beyond descriptive heatmaps. The platform produces reproducible decision-support artefacts, including AOI rankings, heatmaps, and segment-level summaries, grounded in objective behavioural data. By separating the decision signal (gaze) from contextual descriptors (demographics), this work contributes a reusable end-to-end platform for marketing and UX research. It supports choice inference under constrained conditions and segment-level interpretation without demographic priors in the decision mechanism.

## 1. Introduction

Traditional marketing research relies heavily on self-reported data from surveys, interviews, and focus groups to assess consumer preferences, attitudes, and intentions. These instruments are informative and easy to deploy at scale. However, self-report measures are vulnerable to cognitive bias, memory limitations, social desirability effects, and response styles. As a result, they may distort how consumers behave in real time [1]. In fast-moving markets, organisations increasingly need methods that complement declarative reports with direct behavioural evidence of attention and engagement [2].

Advances in eye tracking, computer vision, and machine learning make this possible. Eye tracking provides time-resolved records of where people look, for how long, and in what sequence. It turns visual attention into a measurable behavioural signal [3]. In parallel, facial analysis can estimate affective states or demographic attributes (e.g., age, gender, ethnicity) from video streams. This supports richer segmentation and contextual interpretation of behaviour [4].

Most existing studies in marketing, consumer research, and UX apply these technologies in isolated case studies. They often rely on bespoke setups with limited integration across modalities. Analyses are frequently offline and descriptive, focusing on heatmaps, fixation counts, or group-level comparisons. Fewer works emphasise predictive modelling or reusable decision-support tools that generalise across campaigns and designs [5].

This work addresses three core challenges. First, it tackles bias and inaccuracy in self-reported consumer data. It argues that direct measurements of gaze and facial behaviour can complement, rather than replace, traditional instruments. Second, it targets the need for real-time, objective insight into attention and engagement in realistic marketing and UX scenarios (e.g., packaging shelves, websites, social feeds). Third, it proposes a reusable platform in which gaze-derived behavioural signals support outcome inference (e.g., choice, engagement, task performance). Additional modalities provide contextual information for post hoc interpretation. These aims lead to the following research questions (RQs):

- RQ1. How can a single multimodal framework be designed and implemented to support comparable eye tracking studies across heterogeneous marketing and UX scenarios (e.g., packaging shelves, shopper tasks, websites, social feeds, concept boards) under a unified instrumentation and analysis pipeline?
- RQ2. To what extent does linking AOI-level gaze metrics with ML-based demographic estimates enable stratified, segment-level interpretation of observed choice outcomes, compared with gaze-only reporting?
- RQ3. How can this multimodal, gaze-driven framework be instantiated as an end-to-end, transferable prototype that produces reusable decision-support artefacts (e.g., heatmaps, AOI rankings, segment-specific indicators) applicable to new campaigns and designs?

To address these questions, we developDeepVisionAnalytics, a framework that combines eye tracking with ML-based facial analysis to provide an integrated view of visual attention and demographic context. The system is conceived as a general-purpose platform that can be instantiated across multiple marketing and UX settings, rather than as a one-off experimental setup.

In addition to thematic and empirical reviews, recent bibliometric analyses have examined the evolution of eye tracking research across disciplines, highlighting sustained growth, increasing methodological diversification, and the consolidation of eye tracking as a core tool in behavioural, cognitive and applied research domains (e.g., marketing and UX). These bibliometric perspectives complement narrative and PRISMA-based reviews by mapping publication trends, influential outlets, and methodological shifts in the field [6,7].

### 1.1. Theoretical Positioning in Consumer Behaviour

This study is grounded in established theories of consumer behaviour and marketing, particularly research on visual attention, information processing, and decision heuristics. A substantial body of literature demonstrates that visual attention plays a central role in guiding consumer information acquisition; however, attention alone does not necessarily imply preference or positive evaluation. Attention may be driven by visual salience, novelty, task constraints, or cognitive load, independently of liking or choice.

Accordingly, the present work does not equate gaze with preference per se. Instead, gaze is treated as an observable behavioural signal whose interpretive value depends on context, task structure, and decision immediacy. In constrained, visually driven tasks with limited alternatives and low interaction friction, attention can approximate revealed choice. In more complex or exploratory contexts, however, attention should be interpreted as one component within a broader decision-making process rather than as a direct proxy for preference.

The theoretical gap addressed by this work lies not in proposing new consumer behaviour constructs, but in operationalising existing theory through a reusable, multimodal measurement framework. While prior research has extensively theorised the role of attention in consumer decision-making, fewer studies provide end-to-end systems that translate these concepts into real-time, multimodal analytics capable of supporting practical marketing and UX decisions.DeepVisionAnalytics bridges this gap by linking theory-informed behavioural assumptions to a configurable, data-driven platform.

### 1.2. Structure of the Article

Section 2 describes the methodology used to identify and select 28 Scopus-indexed eye tracking studies in marketing, consumer research, and UX. It also outlines their classification into three topics. Section 3 synthesises the main empirical findings across packaging/shopper, advertising, and UX/UI strands. It then highlights the gaps that motivate a multimodal, platform-based approach. Section 3.1 positions the contribution of this work relative to those strands. Section 4 presents the overall architecture of the DeepVisionAnalytics model, combining eye tracking, computer vision, and multitask machine learning. Section 5 details the prototype implementation, including the gaze-tracking pipeline, the facial recognition and demographic inference module, and real-time multimodal data fusion. Section 6 reports quantitative results for the demographic models, gaze-based choice prediction, and usability tests. Section 7 discusses implications for marketing and UX practice and situates the contributions within the literature. Section 8 outlines limitations and directions for future research. Section 9 concludes the paper.

## 2. Methodology

According to iMotions [8], eye tracking research typically falls into three broad categories: (i) scientific research, (ii) marketing and user research, and (iii) industry and human performance.

In this study, we focus explicitly on the marketing and user research strand—which includes shopper and packaging research, advertising effectiveness, and user experience (UX) evaluation—and on work that relates gaze patterns to consumer choice and decision-making [9,10]. To operationalise this focus, we ran an advanced search in Scopus using a TITLE-ABS-KEY query that required, simultaneously, the following: (i) at least one eye tracking or gaze-related term (“eye-tracking”, “eye tracking”, “gaze-tracking”, “gaze tracking”, “gaze estimation”, “visual attention”, “eye movements”, “eye trackers”); (ii) at least one face-analysis term (“face recognition”, “face-recognition”, “face emotion recognition”, “facial expressions”); and (iii) at least one marketing or user research term (marketing OR “user experience” OR UX OR “shopper research” OR shopper OR “packaging research” OR packaging OR advertising OR “advertising effectiveness”). The search was limited to peer-reviewed journal articles published between 2016 and 2024; no language restrictions were applied.

For transparency and reproducibility, the exact Scopus advanced search query was as follows:

TITLE-ABS-KEY (("eye-tracking" OR "eye tracking" OR "gaze-tracking"

OR "gaze tracking" OR "gaze estimation" OR "visual attention"

OR "eye movements" OR "eye trackers")

AND ("face recognition" OR "face-recognition"

OR "face emotion recognition" OR "facial expressions")

AND (marketing OR "user experience" OR UX

OR "shopper research" OR shopper OR "packaging research"

OR packaging OR advertising OR "advertising effectiveness"))

AND (LIMIT-TO (DOCTYPE, "ar")) AND (LIMIT-TO (PUBYEAR, 2016)

OR LIMIT-TO (PUBYEAR, 2017) OR LIMIT-TO (PUBYEAR, 2018)

OR LIMIT-TO (PUBYEAR, 2019) OR LIMIT-TO (PUBYEAR, 2020)

OR LIMIT-TO (PUBYEAR, 2021) OR LIMIT-TO (PUBYEAR, 2022)

OR LIMIT-TO (PUBYEAR, 2023) OR LIMIT-TO (PUBYEAR, 2024))

AND (LIMIT-TO (SRCTYPE, "j")) AND (LIMIT-TO (PUBSTAGE, "final"))

AND (LIMIT-TO (OA, "all" ))

### 2.1. Search and Selection Process

**Figure 1:**
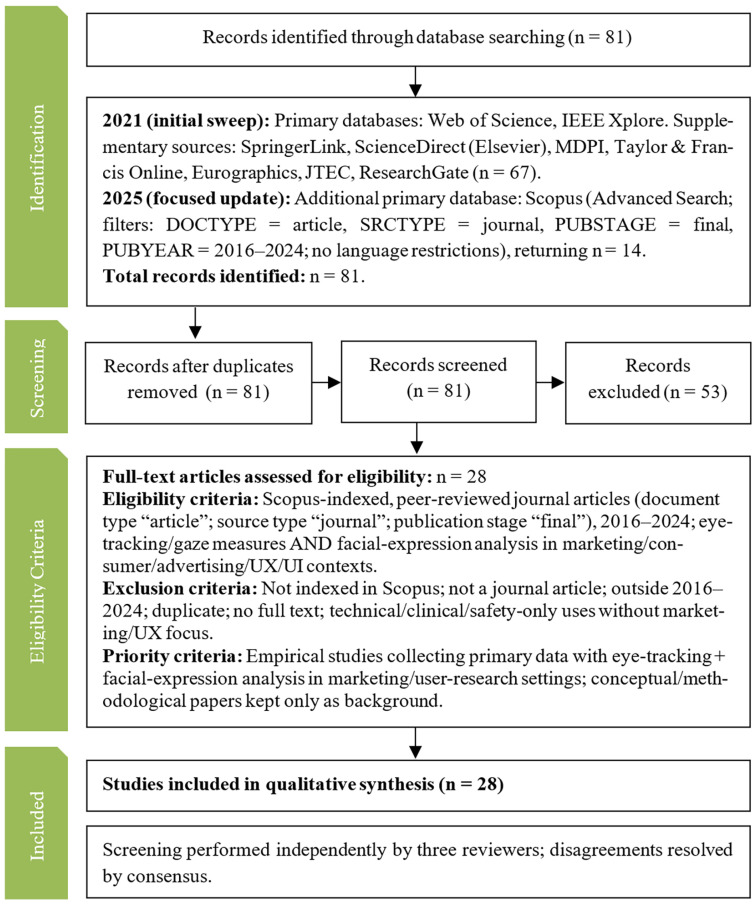
PRISMA flow diagram summarizing the results of the search and selection processes.

The overall search and selection procedure followed PRISMA guidelines [11] and is summarised in Figure 1. The process unfolded in two stages.

First, an initial sweep conducted in 2021 queried multiple primary databases (Web of Science and IEEE Xplore) and complementary sources (SpringerLink, ScienceDirect (Elsevier), MDPI, Taylor & Francis Online, Eurographics, JTEC, and ResearchGate), yielding 67 records. Second, a focused update in 2025 used Scopus as an additional primary database, with the advanced search strategy described above and filters DOCTYPE = article, SRCTYPE = journal, PUBSTAGE = final, and PUBYEAR = 2016–2024, returning 14 records. In total, n=81 unique records were identified.

After removal of duplicates, all 81 records were screened at title/abstract level. This stage led to the exclusion of 53 papers that were clearly out of scope (e.g., technical or clinical applications with no marketing, consumer, or UX focus). The remaining 28 papers were retrieved in full text and assessed for eligibility.

Eligibility criteria required that studies (i) were Scopus-indexed, peer-reviewed journal articles; (ii) reported empirical work collecting primary data with eye tracking or closely related gaze measures; (iii) combined eye tracking/gaze with facial expression analysis (e.g., emotion recognition, facial action coding) or other face-based measures; and (iv) were situated in marketing, consumer, advertising, or UX/UI contexts. Exclusion criteria comprised not being indexed in Scopus; not being a journal article; publication outside 2016–2024; lack of full text; or focusing exclusively on technical, clinical, or safety-related uses with no consumer or UX angle.

Priority was given to empirical studies that collected primary data with both eye tracking and facial expression analysis in marketing or user research settings. Conceptual or purely methodological papers were retained only as background. Screening and eligibility assessments were performed independently by three reviewers, and all disagreements were resolved by consensus.

Following the PRISMA-based procedure [11] (Figure 1), the database searches returned 81 records. After duplicate removal and title/abstract screening, 53 records were excluded. Twenty-eight full text journal articles met the eligibility criteria and were retained for qualitative synthesis. These 28 empirical studies were then grouped into three research topics that reflect the marketing/user research strand of Tobii’s framework: (i) UX/UI, (ii) advertising and digital persuasion, and (iii) packaging and shopper research. Some papers span more than one topic (e.g., e-commerce work relevant to both UX and advertising); in such cases, each paper is assigned to the topic that best reflects its primary research goal. Conceptual or methodological papers (e.g., process-tracing or bibliometric overviews) are cited as background but are not counted within the set of 28 empirical studies.

### 2.2. Research Topics and Classification

This classification summarises how the 28 approved empirical studies are distributed across three topics—UX/UI, advertising and digital persuasion, and packaging/shopper research (Table 1). It provides the basis for the detailed synthesis in Section 3, with multi-topic papers assigned to the topic that best reflects their primary research goal.

## 3. Synthesis of Findings Across Three Clusters (Packaging, Advertising, UX/UI)

This section synthesises 28 empirical, Scopus-indexed studies that apply eye tracking in marketing, consumer, and UX/UI contexts (Table 1). Across these articles, gaze data consistently indicate where and when attention is allocated, while, in some cases, additional measures inform how users experience stimuli. Taken together, the evidence shows that attention is necessary but not sufficient: visual salience, affective engagement, and task context jointly shape evaluation and choice, rather than gaze alone.

### 3.1. Packaging and Shopper Research

Packaging and in-store studies [1,2,5,28,29,30,31,32,33,34,35,36] converge on three robust results. First, visually salient elements such as logos, product images, colour blocks, and strong contrasts capture both early and sustained attention, whereas fine-print claims, dense text, and mandatory information (for example, nutritional tables and legal warnings) are systematically under-attended. Second, when on-pack claims or labels do influence choice, this typically occurs when they are congruent with prior expectations and category beliefs, rather than simply because additional information is present. Third, multiple experiments document that attention does not equate to liking: highly fixated packages are not always better liked, recalled, or chosen, and similar scan paths can correspond to different evaluations, especially when affective or contextual factors are taken into account.

### 3.2. Advertising and Digital Persuasion

In advertising and broader digital persuasion settings [23,24,25,26,27], eye tracking results show that emotional narratives, human faces (including influencers), and rich imagery are effective at attracting gaze and maintaining engagement with the message. However, this attentional pull does not automatically produce better comprehension or persuasion. Intrusive formats (for example, pop-up-like intrusions), visually cluttered layouts, or emotionally intense content can reduce message processing, increase irritation, and, in some cases, impair recall or understanding of risks. Across banners, video advertising, influencer campaigns, and other digital formats, outcomes depend on how and when visual elements compete with critical text and on whether viewing is goal-directed or more exploratory, rather than on attention alone.

### 3.3. UX and UI

For UX and interface evaluation, the analysed empirical studies [12,13,14,15,16,17,18,19,20,21,22] show that eye tracking is a robust diagnostic tool to locate friction points and to verify whether actual use matches design intentions. Across websites, social feeds, games, narrative visualisations, and interactive tools, gaze data repeatedly highlight ignored buttons, cluttered menus, misleading affordances, and distracting visuals. Some experiments indicate that adding game elements or richer interaction can increase stimulation and engagement without harming task performance, provided that core tasks remain clear [21]. Other work demonstrates that comparatively inexpensive eye trackers, when combined with careful calibration and experimental control, still yield diagnostically useful data, supporting more routine integration of gaze in UX workflows [12,19,20]. A smaller set of studies explores gaze-driven adaptations and narrative personalisation [14,17], but most contributions remain descriptive and post hoc rather than fully closed-loop.

### 3.4. Positioning and Current Work Contribution

Against this backdrop of mostly single-study, single-stimulus projects focused on specific product categories or interfaces such as orange juice, chocolate, beer, travel websites, social feeds, online reviews, or individual games [1,5,12,15,18,21,22,26,35], the present work proposes DeepVisionAnalytics as a reusable platform that supports multiple marketing and UX scenarios (packaging shelves, shopper tasks, websites, feeds, concept boards, and other interfaces) under a single instrumentation and analysis pipeline. Instead of producing isolated diagnostics per study, the system is designed for repeated experiments, reconfigurable stimuli and cumulative learning across contexts.

In terms of modalities and data fusion, this work goes beyond what is reported in the existing empirical literature. Existing studies typically combine gaze with self-report and, in a subset of cases, with basic physiological measures [2,17,29,30,33]. None of the 28 empirical papers train or deploy dedicated machine learning models to infer age, gender, and ethnicity as an explicit demographic layer within a consumer or UX pipeline. DeepVisionAnalytics integrates eye tracking with automatic demographic facial analysis, capturing time-aligned AOI metrics (…) while demographic estimates are linked post hoc as contextual metadata to enable stratified, segment-level interpretation of gaze-derived outcomes.

At the level of instrumentation and ecological validity, several reviewed studies rely on commercial suites or lab-specific tools tailored to a single project (for example, iMotions-based setups in packaging and shopper research, bespoke platforms for UX, and narrative visualisation). By contrast, this work implements an end-to-end, transferable stack—capture, synchronisation, analytics, and reporting—that yields exportable heatmaps, per-user and per-image statistics, “most-viewed” rankings, and configurable AOIs across study types, while supporting quasi-realistic tasks such as multi-product shelves, live/social feeds, and interactive interfaces.

To make this platform-level distinction explicit, Table 2 contrasts DeepVisionAnalytics with representative commercial research suites.

From an analytical perspective, most of the 28 articles rely on classical inferential statistics to compare conditions using gaze metrics, self-reports, and occasional physiological measures, while technical contributions often treat eye tracking primarily as ground truth rather than as input to predictive systems [17,31,32,34]. By contrast, DeepVisionAnalytics reframes the problem at the system level: candidate choice is inferred exclusively from gaze-derived behavioural signals, while demographic attributes are collected independently and linked post hoc as contextual metadata. This design enables stratified and segment-level interpretation and validation of observed outcomes (e.g., choice, engagement, task success) without using demographic variables as decision features or predictors in the choice inference process.

Finally, while most reviewed studies end with significance tests and qualitative design suggestions [5,18,22,23,24,27,28], this work delivers a concrete decision-support tool. The platform generates ranked AOIs, estimated lifts in willingness to buy and recall, segment-specific insights by age, gender and ethnicity, and exportable summaries (heatmaps, tables, and rankings) that practitioners can apply directly to new campaigns and designs. In doing so, it moves beyond isolated case studies or purely methodological contributions and provides an extensible DeepVisionAnalytics prototype capable of replicating and extending prior findings across multiple marketing and UX scenarios.

To summarise the contribution of the present work relative to prior empirical studies reviewed in Section 3, Table 3 contrasts typical characteristics of the literature with the proposed platform.

### 3.5. Conceptual Framework Linking Attention, Context, and Consumer Outcomes

**Figure 2:**
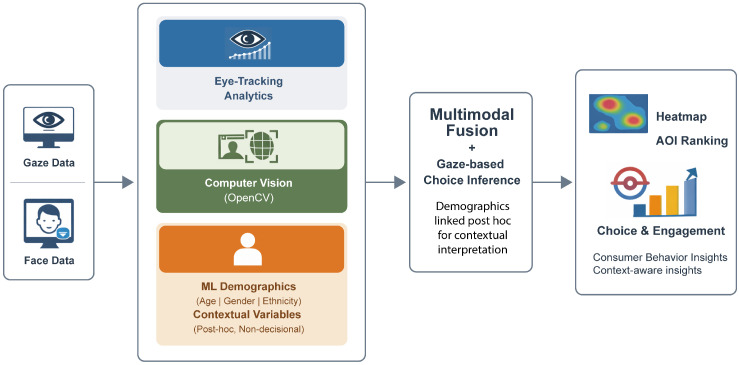
Conceptual framework linking visual attention (AOI metrics), demographic context, and consumer outcomes under explicit behavioural assumptions.

Figure 2 presents the conceptual framework underpinning this study. The framework formalises the roles of visual attention and demographic context within the proposed multimodal analytics platform, explicitly distinguishing between behavioural signals used for choice inference and contextual variables used for post hoc interpretation. In doing so, it provides a clear theoretical bridge between consumer behaviour research and the system architecture illustrated in Figure 2.

Visual attention constitutes the primary behavioural signal in the framework and is operationalised through AOI-level gaze metrics (e.g., time to first fixation, dwell time, revisits). These metrics capture where and how consumers allocate perceptual resources during interaction with stimuli and serve as the sole input for gaze-based choice inference under the experimental conditions considered. Attention is thus treated as an observable proxy for information acquisition rather than as a direct or universal indicator of preference.

Demographic inference (age, gender, ethnicity), obtained via ML-based facial analysis, is introduced as a contextual and segment-level variable. As reflected in Figure 2, demographic attributes are linked to gaze-derived outcomes post hoc and do not participate in the decision mechanism itself. Their role is interpretative rather than predictive: demographics enable stratified analyses and segment-level comparisons of gaze behaviour and inferred outcomes, but they do not determine individual choice or engagement.

Consumer outcomes—such as inferred choice, engagement indicators, or task success—are therefore modelled as higher-level constructs driven primarily by visual attention, with demographic context moderating interpretation at the analytical stage. The framework makes explicit that gaze-based inference approximates observed choice only under constrained conditions, such as visually driven tasks with limited alternatives, short decision horizons, and low cognitive load. In more complex, exploratory, or affect-laden decision contexts, attention should be interpreted as one component within a broader decision-making process rather than as a direct proxy for preference or liking.

By explicitly separating decision-driving signals from contextual descriptors, the conceptual framework clarifies the scope, assumptions, and limits of the proposed approach and guides the interpretation of results generated by the DeepVisionAnalytics platform.

Visual attention is operationalised through AOI-level gaze metrics (e.g., time to first fixation, dwell time, revisits), capturing where and how consumers allocate perceptual resources during interaction with stimuli. These metrics represent observable behavioural signals related to information acquisition rather than direct indicators of preference.

Demographic inference (age, gender, ethnicity) is introduced as a contextual and segment-level variable rather than a deterministic predictor. Demographics moderate the interpretation of attention patterns by enabling comparative analysis across consumer segments, but they do not directly determine individual choice or evaluation.

Consumer outcomes—such as choice, engagement, or task success—are modelled as higher-level constructs that emerge from the interaction between attention patterns and contextual factors. Importantly, the framework makes explicit that gaze-based inference approximates preference only under constrained conditions, such as visually driven tasks with limited alternatives, short decision horizons, and low cognitive load. In more complex, exploratory, or affect-laden decision contexts, attention should be interpreted as one component within a broader decision-making process rather than as a proxy for liking or preference.

By making these assumptions explicit, the conceptual framework clarifies the scope and limits of the proposed approach and guides the interpretation of results generated by the DeepVisionAnalytics platform.

## 4. Model Architecture

We propose an integrated model that combines eye tracking (ET), OpenCV-based computer vision (CV), and machine learning (ML) for facial recognition (FR) and demographic classification. By capturing gaze data and facial features in real time, the system produces objective, data-driven insights into consumer–product interactions, where gaze-derived signals drive choice inference and demographic estimates serve exclusively as contextual descriptors for post hoc interpretation.

The ET stream provides gaze positions, fixation durations, and attention heatmaps. In parallel, CV components (OpenCV) handle face detection, bounding-box generation, and feature extraction. ML models—convolutional neural network (CNN) heads for age (regression), gender (binary classification), and ethnicity (multi-class classification)—convert these features into demographic predictions that can be joined to gaze events for analysis.

**Figure 3:**
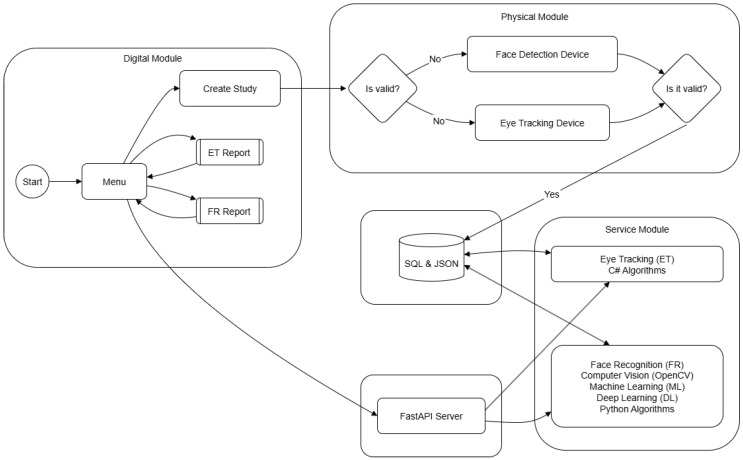
Overall system architecture organised into five functional modules.

The architecture is organised into five modules (Figure 3). The Digital module creates and manages studies, user interfaces, and result centralisation. The Physical module integrates the Tobii eye tracker and a webcam for real-time capture. The Connection module (FastAPI) links hardware and software with low-latency messaging. The Data module persists structured and semi-structured records in SQL Server and JSON stores. The Service module runs FR/ML pipelines (OpenCV, AI/ML) and exposes their outputs to the application. A user/manager interaction surface consumes these services for monitoring and reporting.

End-to-end, the Digital and Physical modules feed events through the Connection layer into the Service and Data modules, where FR/ML inference and storage occur; results are then shown to operators for decision-making.

### Technologies

The prototype combines a set of mature, widely used technologies, each chosen for a specific role in the pipeline. Python 3.8.19 is used as the main backend language because it offers a large ecosystem for computer vision, machine learning, and data processing. Within this ecosystem, OpenCV handles image pre-processing (resizing, normalisation, colour-space conversions), real-time face detection, and basic feature extraction from webcam frames. On top of this, TensorFlow is used to implement and train the convolutional neural network (CNN) for facial recognition and demographic classification, providing GPU acceleration, model export, and integration with standard deep learning workflows. LabelImg is employed during the data preparation phase to annotate bounding boxes around faces, generating the labelled datasets required to train and validate the detection and recognition models.

On the application side, C# and UWP (Universal Windows Platform) support the graphical user interface and hardware integration under Windows. UWP provides direct access to devices such as the eye tracker and webcam, as well as high-frequency timers and rendering primitives needed for smooth real-time visualisation of gaze and facial analysis outputs. The ET/FR backend written in Python is exposed to the UWP client through FastAPI, which offers a lightweight HTTP/REST interface with low latency, allowing gaze samples and video frames to be sent to the server and predictions to be returned in near real time.

For data management, the system uses a hybrid storage strategy. The SQL Server stores structured entities such as studies, users, sessions, and aggregated metrics (e.g., per-image statistics, AOI-level indicators), enabling relational queries and integration with existing analytics tools. In parallel, JSON files and JSON columns are used to persist semi-structured information such as raw event logs, gaze traces, configuration parameters, and diagnostic outputs, which would be cumbersome to normalise fully into relational tables. Together, this stack provides computational efficiency for real-time processing, flexibility to evolve the models and interfaces, and robustness for storing and analysing consumer behaviour data at session and study levels.

## 5. Prototype Implementation

**Figure 4:**
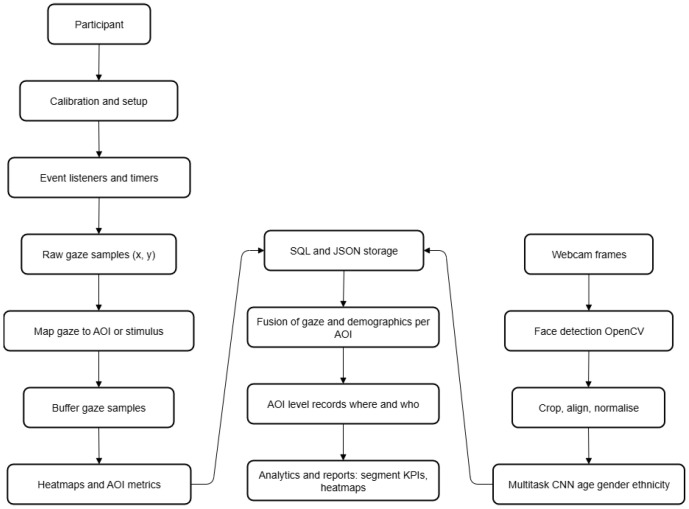
Extended runtime pipeline integrating eye tracking, facial analysis, and multimodal fusion.

The prototype integrates eye tracking and facial analysis to generate real-time behavioural evidence. The system tracks gaze (e.g., fixation durations and gaze positions) to infer candidate choice under constrained experimental conditions, and independently estimates demographic attributes (age, gender, and ethnicity) via an ML-based facial analysis module. Demographic attributes are not used to drive choice inference; instead, they are linked post hoc to gaze-derived outcomes to support stratified and segment-level interpretation. Unlike self-reported surveys, which are often affected by recall errors and social desirability bias, this multimodal pipeline relies on objective measurements of visual attention, complemented by demographic context for analytical interpretation. Figure 4 summarises the implementation pipeline.

Eye tracking branch (left column). From the participant’s perspective, the session starts with a brief calibration and setup stage, after which the eye tracking service configures event listeners and timers that detect when gaze starts, moves, or stops and that define the sampling frequency. These listeners produce a continuous stream of raw gaze samples (x,y) in screen space. Each sample is then used to map gaze to the current AOI or stimulus by checking whether its coordinates fall inside predefined container bounds (product images, interface elements, etc.). To avoid excessive I/O, gaze samples are first buffered in memory and only periodically written to persistent storage. The buffered stream is then aggregated into heatmaps and AOI metrics (e.g., dwell time, visits, time to first fixation), which are stored in the SQL and JSON data module for subsequent analysis.

Facial recognition and demographic inference branch (right column). In parallel, a webcam captures frames of the participant’s face. These frames are fed to an OpenCV-based module for face detection, after which detected faces are cropped, aligned, and normalised to a fixed resolution (e.g., 128×128). The normalised crops are passed to a lightweight multitask CNN (age, gender, ethnicity) based on MobileNetV2, which outputs continuous age estimates and categorical gender/ethnicity labels in real time. These predictions are also logged to the SQL and JSON storage layer, keyed by timestamp and participant/study identifiers.

Fusion and AOI-level analytics (central column). Once gaze and demographic streams are stored, the system links demographic estimates to gaze-derived AOI outcomes post hoc, producing stratified AOI summaries (e.g., AOI metrics by age group or gender) for contextual interpretation. On top of these fused records, the analytics layer computes segment-level KPIs and reports—for example, heatmaps and AOI metrics by age group or gender, ranked stimuli (“most viewed” images), and lifts in willingness-to-buy or recall for specific segments. These outputs are then used to generate the visual and tabular reports presented to marketing and UX teams.

Model construction, training, and evaluation (offline stage). The multitask CNN used in the facial recognition branch is trained offline on pre-processed facial images in which faces are detected (Haar cascades and deep learning detectors such as SSD or MTCNN), resized, and normalised. Data augmentation (rotation, brightness changes, horizontal flips, Gaussian noise) improves generalisation, and the dataset is split into training (70%), validation (15%), and test (15%) subsets. The network is optimised with the Adam optimiser and an adaptive learning rate schedule; mean absolute error (MAE) is used as the loss for age regression, binary cross-entropy for gender classification, and categorical cross-entropy for ethnicity. Performance is monitored with MAE/mean squared error (MSE) for age and accuracy, precision, recall, and F1-score for gender and ethnicity, complemented by confusion matrices, error histograms, and scatter plots of true versus predicted age to diagnose systematic biases.

Taken together, these components implement an end-to-end pipeline—capture → synchronisation → analytics → reporting—that supports real-time visualisation (gaze overlays, heatmaps) and offline statistical or ML-based analysis of consumer and user behaviour.

## 6. Results and Analysis

**Figure 5:**
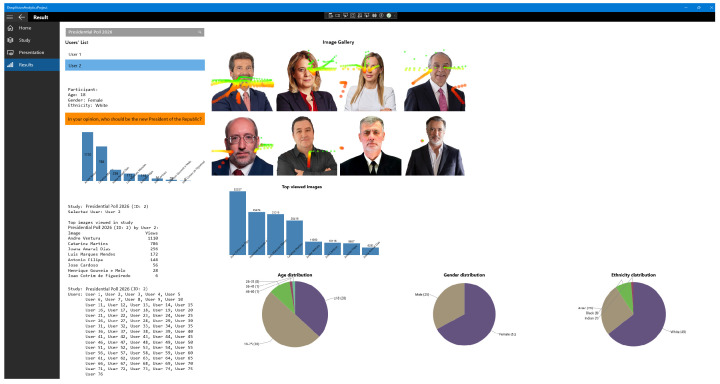
Prototype interface (Result page), aggregating gaze traces, fixations, candidate choices, demographic summaries, and session logs.

Figure 5 shows actual outputs from the prototype collected during participant sessions. The screen aggregates real-time gaze traces and fixations over candidate images, a view/choice bar chart per candidate, a “Top viewed images” ranking, and session logs (IDs, timestamps, actions) for auditability. In parallel, demographic pies (age, gender, ethnicity) are estimated by the FR/ML module and linked to gaze events. Together, these views document what each participant did (where they looked and what they selected), for how long, and who they were (demographic profile), forming the empirical basis for the quantitative analyses reported in Section 6.2, Section 6.3, Section 6.4 and Section 6.5 and Section 6.9. Importantly, gaze data are interpreted here as behavioural indicators of visual attention and information acquisition rather than as direct proxies for preference, and all subsequent analyses should be understood within the scope of a controlled, visually driven experimental setting.

### 6.1. Model Performance and Evaluation

The demographic inference module is implemented as a multitask convolutional neural network (CNN) jointly predicting age (regression), gender (binary classification), and ethnicity (multi-class classification). The model operates on RGB facial crops resized to (128×128×3) and was trained using a fixed, reproducible, and subject-independent data split.

#### 6.1.1. Dataset and Split

The training set comprises 57,193 samples, while the held-out test set contains 14,299 samples. The age target spans integer values up to 67 years in both training and test sets, ensuring consistent label coverage across splits. All reported results are computed exclusively on the held-out test set, which was not used for model fitting, hyperparameter tuning, or early stopping.

#### 6.1.2. Evaluation Protocol and Robustness

The objective of this model is not individual-level prediction, but the extraction of reliable aggregate demographic descriptors to support post hoc, segment-level interpretation of gaze-derived behavioural outcomes. Accordingly, evaluation emphasises held-out performance stability, class balance, and error characteristics rather than optimisation for deployment-critical accuracy.

To assess robustness and replicability, training and evaluation were repeated across R=5 independent runs with different random seeds while preserving the same fixed subject-independent split. Performance is reported as mean ± standard deviation on the held-out test set. In addition, 95% bootstrap confidence intervals (1000 resamples) were computed for the primary metrics (MAE for age estimation; accuracy and macro-F1 for gender and ethnicity classification), providing an estimate of variability across runs without relying on full cross-validation.

Model performance is assessed using task-appropriate metrics: mean absolute error (MAE) and error distributions for age regression; accuracy, precision, recall, and F1-score for gender and ethnicity classification. Subject-independent splits and strict separation between training and test sets ensure the absence of data leakage.

#### 6.1.3. Data Distribution Across Splits

**Figure 6:**
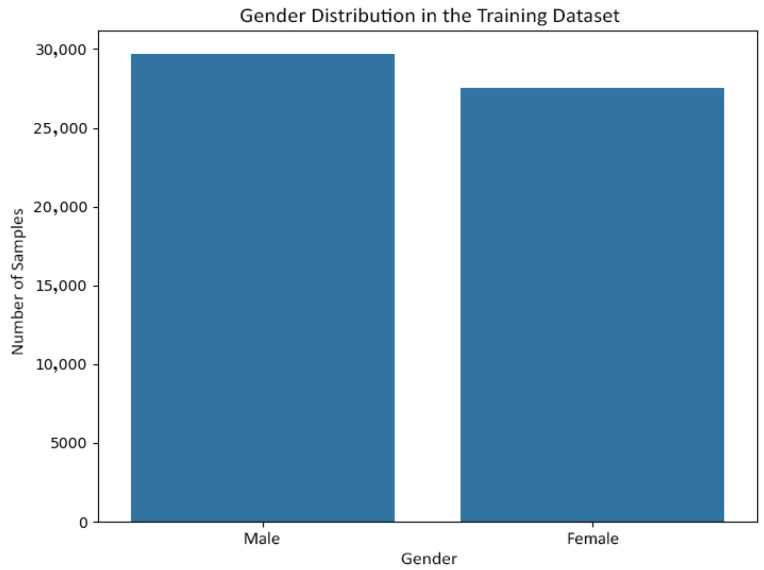
Gender distribution in the training set.

**Figure 7:**
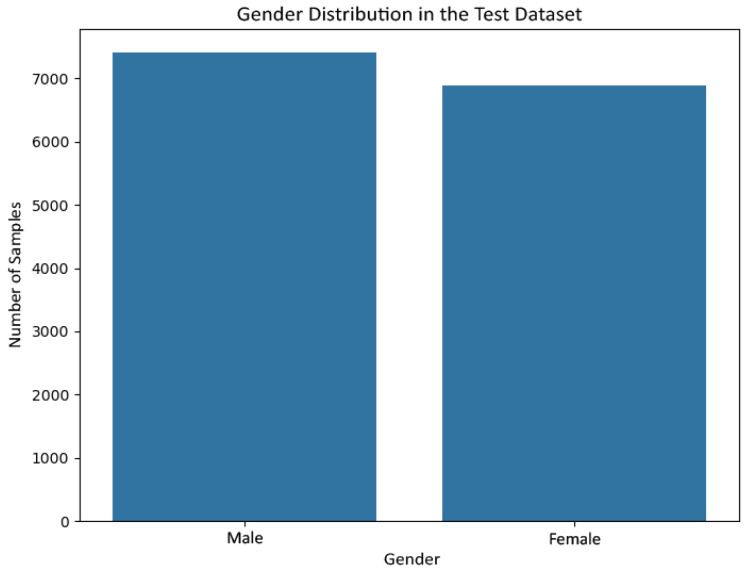
Gender distribution in the held-out test set.

**Figure 8:**
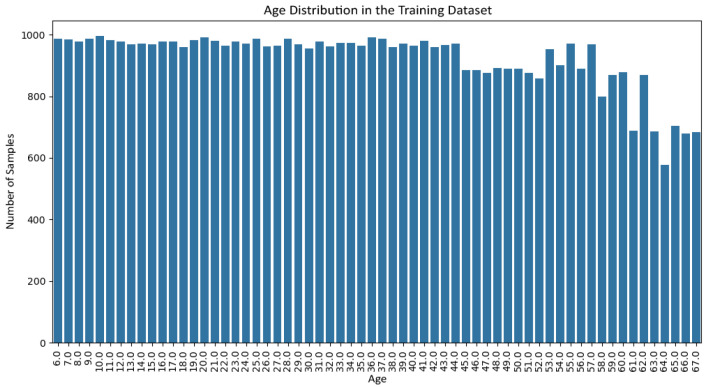
Age distribution in the training set.

**Figure 9:**
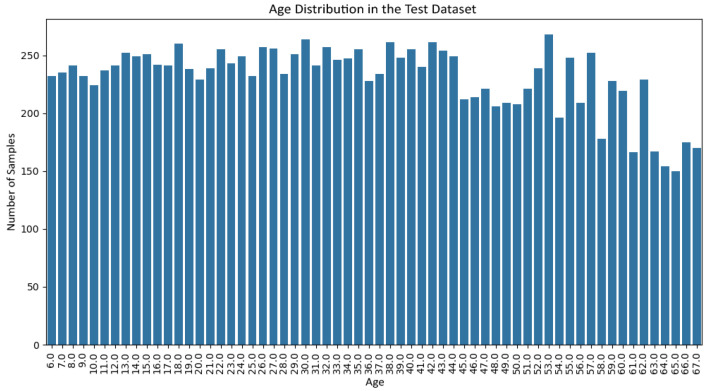
Age distribution in the held-out test set.

**Figure 10:**
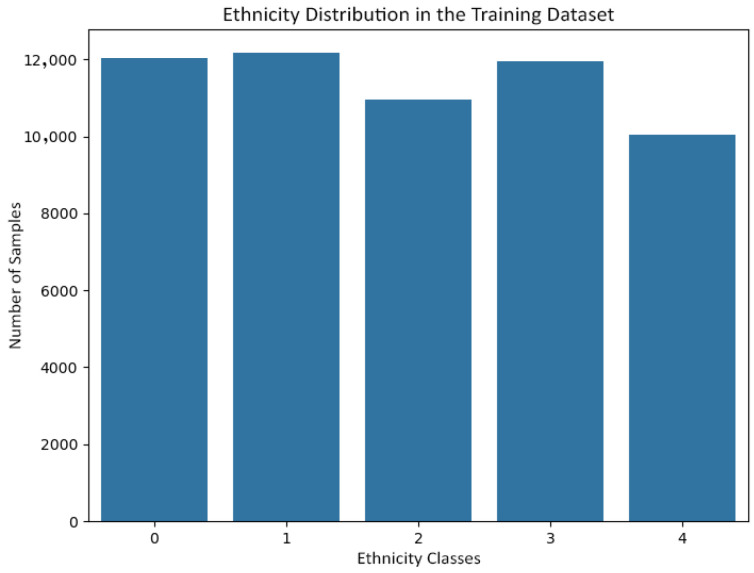
Ethnicity class distribution in the training set.

**Figure 11:**
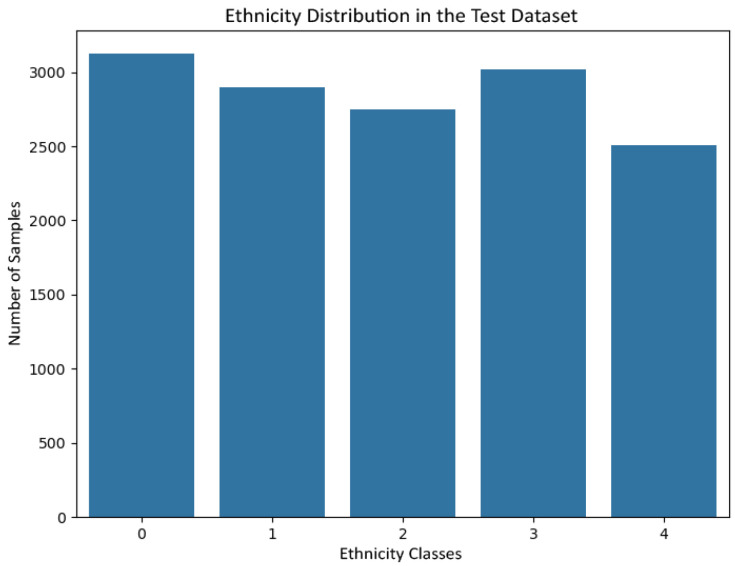
Ethnicity class distribution in the held-out test set.

Figure 6 and Figure 7 show the gender distribution in the training and test sets, respectively, confirming a balanced and comparable class structure across splits. Figure 8 and Figure 9 illustrate the corresponding age distributions, indicating similar coverage across the full age range. Likewise, Figure 10 and Figure 11 confirm consistent ethnicity class proportions between training and test sets. Together, these distributions support the validity of the held-out evaluation and exclude artificial distributional shifts.

#### 6.1.4. Gender Classification

**Figure 12:**
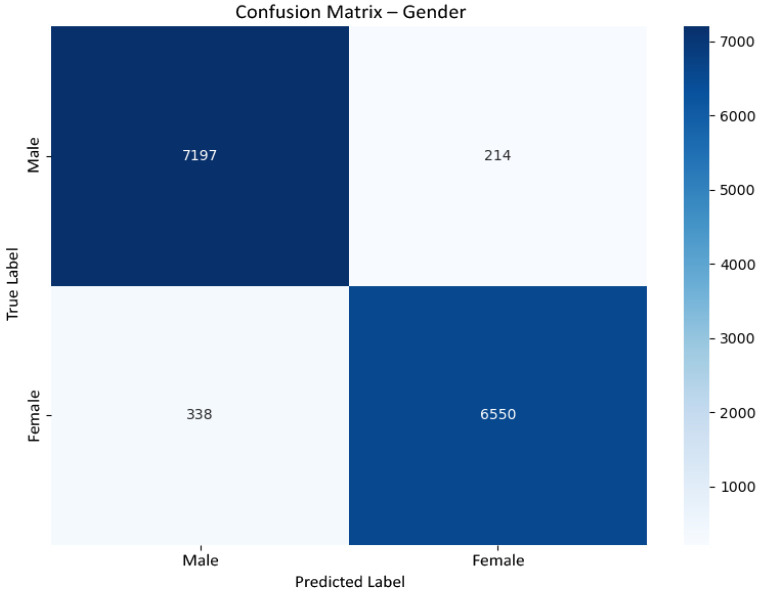
Confusion matrix for gender classification on the held-out test set. True labels are shown on the vertical axis and predicted labels on the horizontal axis.

Figure 12 presents the confusion matrix for gender classification on the held-out test set. The model achieves an overall accuracy of approximately 96%, with balanced precision and recall across classes (macro-F1 ≈0.96). Errors are limited and symmetrically distributed, indicating stable feature extraction and the absence of systematic class bias.

#### 6.1.5. Ethnicity Classification

**Figure 13:**
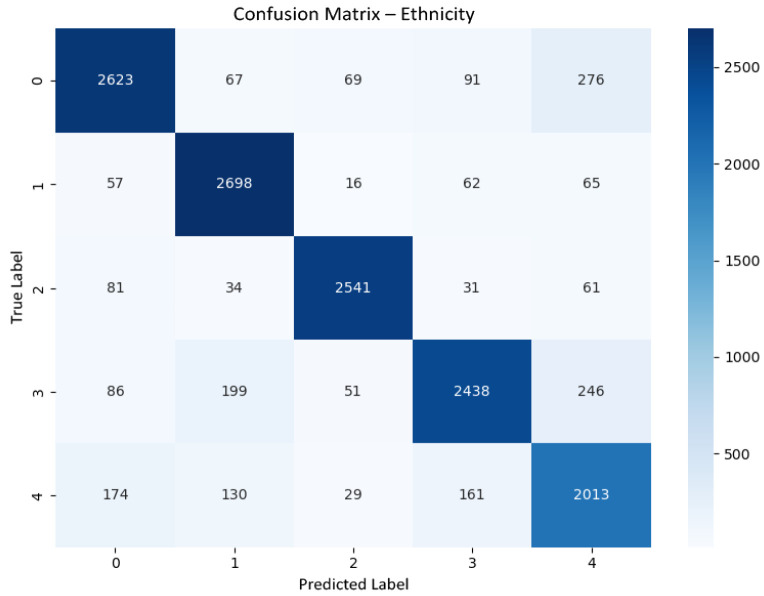
Confusion matrix for ethnicity classification on the held-out test set.

Figure 13 shows the confusion matrix for ethnicity classification on the held-out test set. Strong diagonal dominance is observed, with most misclassifications occurring between visually adjacent or less represented classes, a pattern commonly reported in facial analysis tasks and consistent with dataset-level ambiguity rather than model instability.

#### 6.1.6. Age Estimation

**Figure 14:**
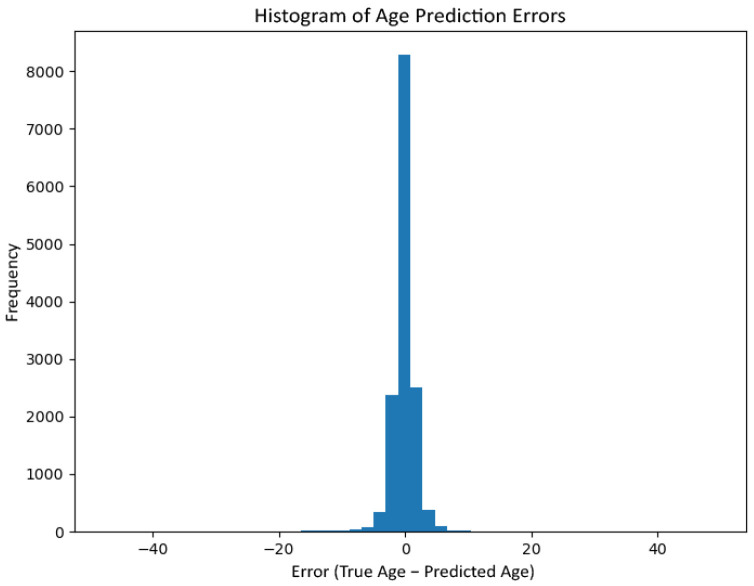
Histogram of age prediction errors (true minus predicted age) on the held-out test set.

**Figure 15:**
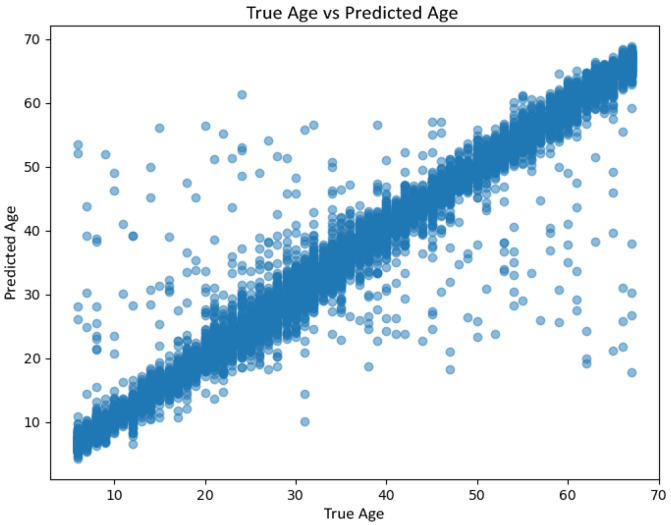
Actual age versus predicted age on the held-out test set. Points close to the diagonal indicate accurate estimation, while dispersion increases for higher age ranges.

Age prediction performance is analysed through error distributions and regression plots rather than a single-point metric. Figure 14 shows the histogram of prediction errors (true minus predicted age), with errors strongly concentrated around zero, indicating low dispersion. Figure 15 plots real versus predicted age, revealing a clear linear relationship with slightly increased variance at higher ages, consistent with known dataset imbalances in older cohorts.

#### 6.1.7. Interpretation and Limitations

Across all tasks, the model demonstrates stable held-out performance and well-behaved error characteristics. Importantly, demographic predictions are not used as decision features for gaze-based choice inference. Instead, they serve exclusively as contextual variables linked post hoc to aggregate behavioural outcomes. Consequently, small residual biases—such as mild age overestimation in older cohorts—do not compromise the validity of the behavioural analyses reported in this work but are explicitly acknowledged to delimit the scope of inference.

### 6.2. Age Validation (Real vs. Predicted)

**Figure 16:**
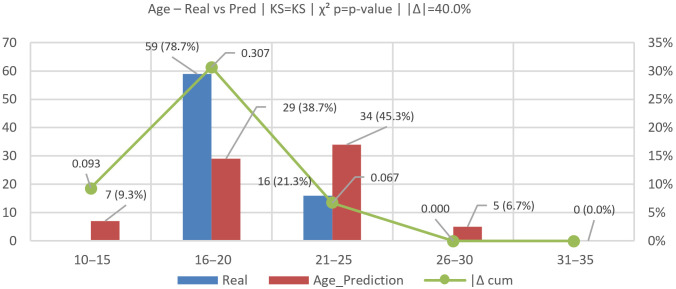
Age (real vs. predicted): empirical distributions and CDFs (including KS and $\chi^{2}$ diagnostics).

**Figure 17:**
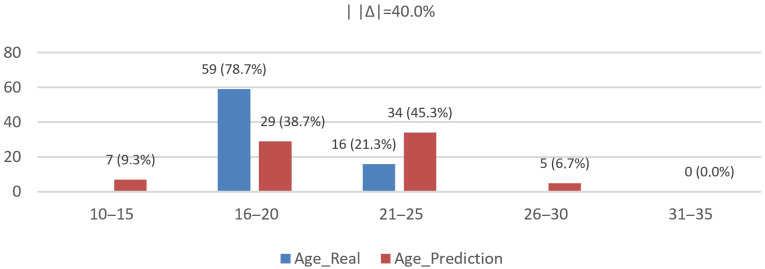
Age (real vs. predicted): histogram of binned distributions (percentage per age bin).

We evaluated age on N=75 records; distributions and diagnostics are shown in Figure 16 and Figure 17 using bins [10–15, 16–20, 21–25, 26–30, 31–35]. The real distribution was [0 (0.0%), 59 (78.7%), 16 (21.3%), 0 (0.0%), 0 (0.0%)], while the model’s predictions were [7 (9.3%), 29 (38.7%), 34 (45.3%), 5 (6.7%), 0 (0.0%)].

**Figure 18:**
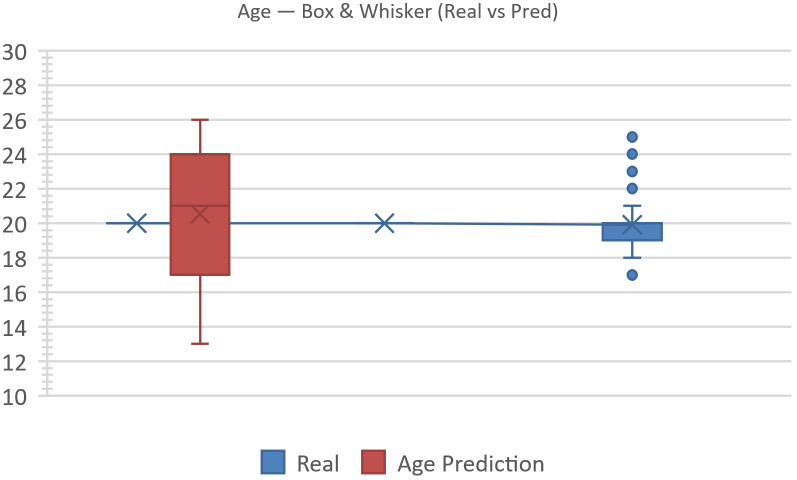
Box-and-whisker comparison of real versus predicted ages.

Figure 18 presents a box-and-whisker comparison of real versus predicted ages. For the real group, the median is 20, IQR = 19–21, whiskers span 18–21, and the mean is 20.00. For the predicted group, the median is 21, IQR = 17–23.5, whiskers span 13–26, and the mean is 20.93. Visually, the predicted box is shifted slightly upward and exhibits longer whiskers, indicating a wider dispersion and heavier tails. The upward shift reflects a systematic overestimation concentrated in the 16–20 bin, while the extended upper whisker captures occasional high predictions.

Concordant with Figure 16, goodness-of-fit tests reveal a notable distributional gap: total-variation distance |Δ|=40.0%; two-sample Kolmogorov–Smirnov (KS) statistic D=0.307 (maximum CDF difference within 16–20), p≈0.0013; and $\chi^{2}\left(3\right)=28.71$, $p\approx 2.6\times 10^{-6}$ with Cramér’s V=0.44. Together, these results confirm a bias that shifts probability mass from 16–20 (real 78.7% → pred 38.7%, −40.0 pp) to 21–25 (21.3% → 45.3%, +24.0 pp), with smaller tails appearing at 10–15 (+9.3 pp) and 26–30 (+6.7 pp).

Actionably, an age-dependent bias correction—for example, a piece-wise-linear adjustment or quantile calibration—can be applied to reduce the upward shift in the 16–20 cohort and trim spurious tails, narrowing the IQR and improving alignment between real and predicted.

#### Post hoc Age Calibration (Preliminary)

To provide a preliminary empirical assessment of the proposed mitigation strategy for the observed age bias, we implemented a lightweight post hoc, bin-wise bias correction applied to the CNN age predictions. Calibration is defined over the same age bins used in the distributional analysis ([10–15, 16–20, 21–25, 26–30, 31–35]). Correction parameters are estimated on the validation split and evaluated on held-out test records (N=75).

Let $\hat{y}$ denote the raw predicted age and $b(\hat{y})$ the age bin to which $\hat{y}$ belongs. For each bin *b*, we estimate a bin-specific bias on the validation set as

$$\delta_{b}=E\left[\hat{y}-y|\hat{y}\in b\right],$$

and apply the following post hoc correction:

$${\hat{y}}^{\;\prime }=\hat{y}-\delta_{b(\hat{y})}.$$

This piece-wise-linear adjustment preserves rank ordering within each bin and does not require model retraining or architectural modification.

The correction specifically targets the dominant mismatch in the 16–20 age cohort, where the predicted mass is substantially lower than the empirical distribution (38.67% vs. 78.67%, corresponding to a −40.0 percentage point deviation).

Table 4 reports distributional alignment metrics and mean absolute error (MAE) before and after calibration. Prior to correction, the maximum absolute bin-level deviation is $\max_{b}\left|\Delta_{b}\right|=40.0$ pp and the Kolmogorov–Smirnov statistic is D=0.307, indicating a pronounced cumulative distributional discrepancy. After calibration, MAE increases from 3.35 to 4.6 years on the test set; moreover, distributional alignment deteriorates, with the maximum bin deviation rising to 57.3 pp and the KS statistic increasing to D=0.573.

This preliminary experiment therefore illustrates that simple bin-wise bias shifts, while intuitive, are insufficient to jointly optimize point-wise accuracy and population-level distributional fidelity, motivating the need for more distribution-aware calibration strategies.

### 6.3. Ethnicity Validation (Real vs. Predicted)

**Figure 19:**
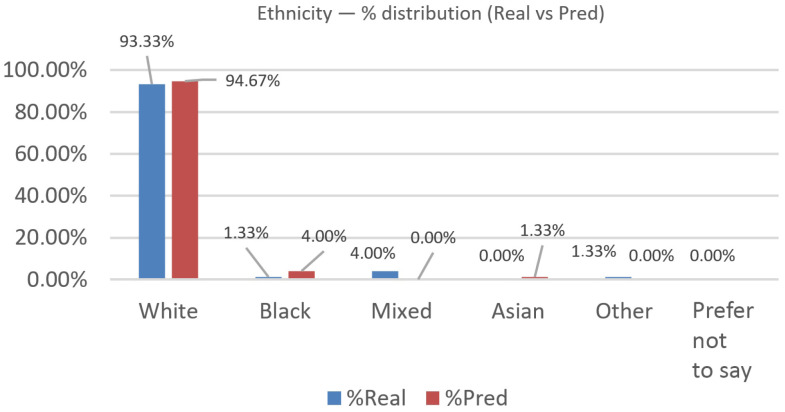
Ethnicity (real vs. predicted): distribution across categories (percentage).

**Figure 20:**
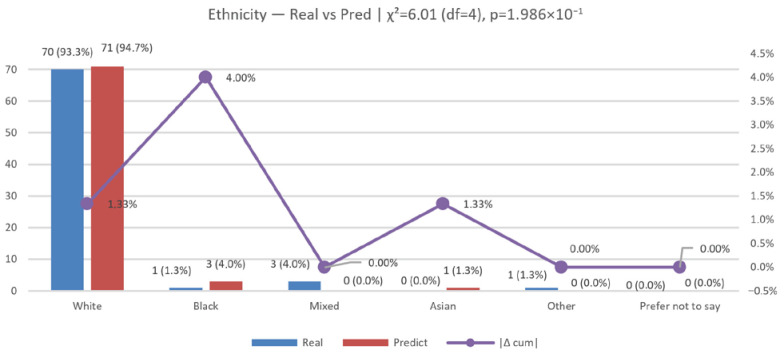
Ethnicity (real vs. predicted): cumulative CDF and cumulative |Δ|.

We evaluated N=75 cases across the categories White, Black, Mixed, Asian, and Other (“Prefer not to say” was 0 in both groups and excluded from $\chi^{2}$); see Figure 19 and Figure 20 for the percentage distribution, per-class $\chi^{2}$ contributions, and cumulative |Δ|.

The observed distributions were as follows: real—White 70 (93.3%), Black 1 (1.3%), Mixed 3 (4.0%), Asian 0 (0.0%), Other 1 (1.3%); predicted—White 71 (94.7%), Black 3 (4.0%), Mixed 0 (0.0%), Asian 1 (1.3%), Other 0 (0.0%). Per-class deltas (Pred − Real, percentage points) were White +1.3, Black +2.7, Mixed −4.0, Asian +1.3, Other −1.3. The overall distributional gap was |Δ|=5.33% (total-variation distance).

A 2×5 chi-square test yielded $\chi^{2}\left(4\right)=6.01$, p=0.199; Cramér’s V=0.20 (small effect; n=150 across both rows), indicating no statistically significant difference between real and predicted distributions at the 5% level.

Overall differences are modest, but there is a clear pattern: under-prediction of Mixed (−4 pp) and over-prediction of Black/Asian (+2.7 pp and +1.3 pp, respectively), with a slight surplus for White (+1.3 pp). This suggests training imbalance.

### 6.4. Gender Validation (Real vs. Predicted)

**Figure 21:**
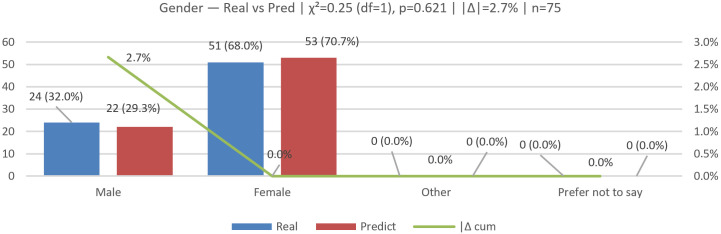
Gender (real vs. predicted): bar comparison of counts.

**Figure 22:**
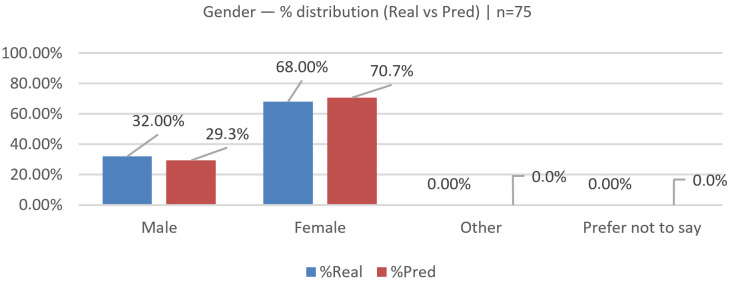
Gender (real vs. predicted): distribution in percentage.

**Figure 23:**
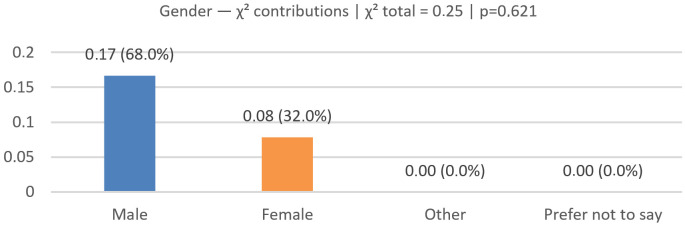
Gender (real vs. predicted): per-class contributions to $\chi^{2}$.

We evaluated N=75 across the categories Male and Female (Other/“Prefer not to say” were zero in both groups and omitted); see Figure 21, Figure 22 and Figure 23 for the bar comparison, percentage distribution, and $\chi^{2}$ contributions.

The observed distributions were as follows: real—Male 24 (32.0%), Female 51 (68.0%); predicted—Male 22 (29.3%), Female 53 (70.7%). Per-class deltas (Pred − Real, percentage points) were Male −2.7 and Female +2.7. The overall distributional gap was |Δ|=2.7% (total-variation distance).

A 2×2 chi-square test yielded $\chi^{2}\left(1\right)=0.25$, p=0.621; Cramér’s V≈0.04 (very small effect; n=150 across both rows), indicating no statistically significant difference between real and predicted distributions.

The model closely matches the real distribution, with a slight tendency to under-predict Male and over-predict Female (±2.7 pp).

### 6.5. Eye Tracking—Category Validation (Real vs. Predicted)

**Figure 24:**
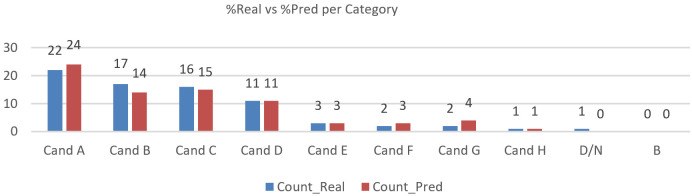
Candidate choice inferred from gaze under a constrained task: real vs. predicted distributions by category.

**Figure 25:**
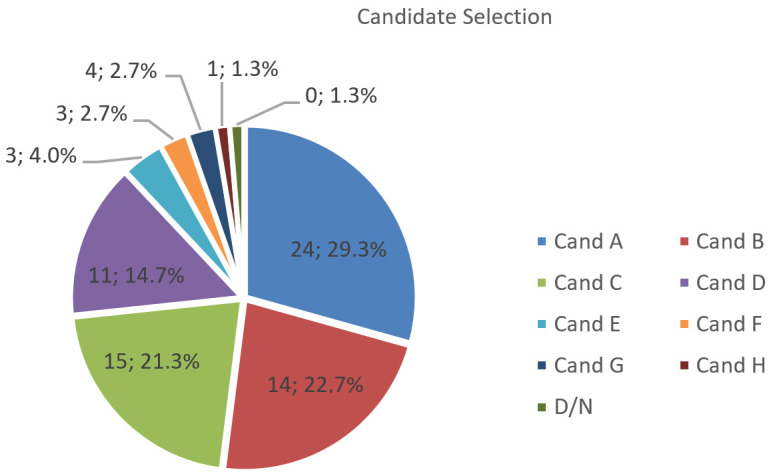
Candidate selection: distribution of choices across the election pool.

**Figure 26:**
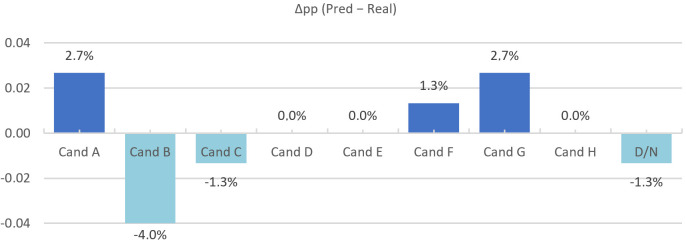
Candidate-level differences (Pred−Real, percentage points) for gaze-based predictions.

We evaluated N=75 trials across the categories Candidate A–H and “Don’t know”. Class-level agreement is summarised in Figure 24, Figure 25 and Figure 26 (per-category bars, election pool pie, and Δpp), and the full confusion structure is shown in Table 5 (Real × Pred with column totals). Overall accuracy is 64/75=85.33%. For the main classes with larger support (row totals ≥10), recall is high for A (20/22 = 90.91%), C (15/16 = 93.75%), and D (10/11 = 90.91%), but lower for B (13/17 = 76.47%). Smaller classes show more variability: E (2/3 = 66.67%) and F (1/2 = 50.00%), while G (2/2) and H (1/1) reach 100% recall but with very low support; “Don’t know” has 0/1. Column-wise precision for the main predicted classes is A (20/24 = 83.33%), B (13/14 = 92.86%), C (15/15 = 100%), and D (10/11 = 90.91%), with E (2/3 = 66.67%), F (1/3 = 33.33%), G (2/4 = 50.00%), and H (1/1 = 100%).

Distributionally, the global gap is |Δ|=6.67% (total-variation distance). A 2×9$\chi^{2}$ test yields $\chi^{2}\left(8\right)=2.28$, p=0.971; Cramér’s V=0.12, indicating a small effect and no statistically significant difference between real and predicted distributions. The main residuals are a mild over-allocation to A and G (+2.7 pp each) and an under-allocation to B (−4.0 pp), while D, E, and H align and “Don’t know” is slightly under-estimated (one real “Don’t know” assigned to F).

Interpretation: The gaze-only classifier reproduces the real candidate distribution closely (Figure 25) in this constrained, visually driven task, with strong A/C/D performance and most errors arising from B confusions (notably B → A) and a few leaks into small classes. These results indicate that gaze-based inference can approximate observed choice outcomes under specific task conditions, rather than constituting a general proxy for preference. Candidate-level interest scoring appears well calibrated overall. If required, light per-class correction (for example, class weights or threshold tweaks) can be applied to reduce bias in B and G while preserving stability in the larger classes (A–D).

### 6.6. Stratified Interpretation Enabled by Demographic Contexts

Table 6 summarises the system-level distinction between predictive and descriptive components. The first row reports performance metrics for gaze-only choice inference, which is the only model used for prediction. The second row documents the role of demographic attributes obtained from independently trained ML models, which are used exclusively for post hoc, segment-level interpretation.

Demographic attributes are not used as predictive features for candidate choice inference. They are linked post hoc to gaze-derived outputs to enable stratified, segment-level interpretation of observed choices, without influencing the inferred decision itself.

### 6.7. Usability and Functionality Tests (2 April 2025)

After completing the core workflow, participants filled out a post-task questionnaire composed of two blocks. The A-block (A1–A9) targeted task success and recommendation: A1 asked whether they could create a new marketing study; A2 whether they could add images to that study; A3 whether they received feedback after creating the study; A4 whether feedback confirmed successful image addition; A5 whether they could configure a random image presentation; A6 whether they received feedback about that randomisation; A7 whether they could view eye tracking and face recognition results; A8 whether privacy and security were maintained; and A9 whether they would recommend the system for marketing studies.

The B-block (B1–B18) covered usability and performance: B1 assessed intuitiveness of creating/configuring a study; B2 difficulties importing images; B3 successful close/save to the database; B4 dynamic collection/storage of ET/FR data; B5 correctness of random product presentation; B6 accuracy of real-time reports; B7 accuracy of real-time FR analysis; B8 ease of navigation; B9 clarity of information/instructions; B10 adequacy of time to complete tasks; B11 perception of the random presentation; B12 comprehension of generated reports; B13 difficulties during interaction; B14 whether the system uses open-source tools; B15 seamless synchronisation between eye tracking and face recognition cameras; B16 functioning without an internet connection; B17 response time during study execution; and B18 performance under different user loads.

Qualitative outcomes: All participants completed the workflow. Most reported high satisfaction with intuitiveness, clarity of feedback, and ET↔FR synchronisation; privacy was generally perceived as preserved; no major performance issues were observed across user conditions, indicating robust responsiveness.

**Figure 27:**
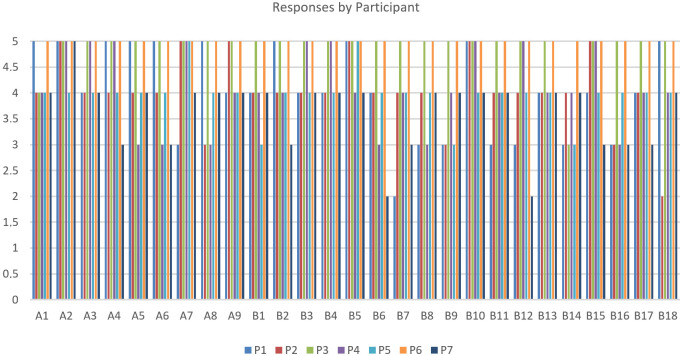
Post-task questionnaire: per-participant responses (Likert 0–5) across items A1–A9 and B1–B18.

**Figure 28:**
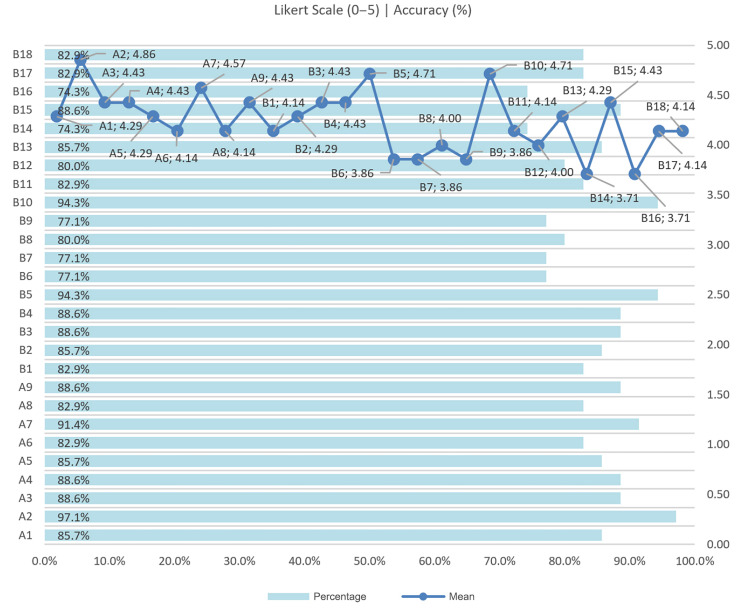
Usability/performance summary: mean score by item (Likert 0–5) and percentage of responses ≥4.

Quantitative outcomes: From the submitted charts, N=7 domain experts (P1–P7) rated 27 items (A1–A9; B1–B18) on a 0–5 Likert scale. The overall mean was ≈4.3–4.4/5, with item scores in the range of 3.7–4.9/5. The strongest items (≈4.7–4.9) were A2, B5, B10, and B15; lower, though still positive (≥3.7), were B16 (≈3.7), B9 (≈3.9), and B12 (≈4.0). The share of positive responses (≥4) was ∼71–94% per item. Given the small usability panel (N=7), the reported Likert means should be interpreted as indicative of pilot usability rather than statistically generalisable estimates, despite the expert nature of the participants. Bottom line: Performance and usability are high; the clear watchpoint is B16 (offline operation), which trails the rest and warrants additional testing and hardening. Per-participant responses in Figure 27, and aggregate results appear in Figure 28 (item means and %≥4).

### 6.8. A/B Test: Paper Ballot vs. Gaze (ET) with Automatic Demographics (2 October 2025)

We conducted an A/B test to assess presidential candidate choice under two conditions. In condition (A), participants completed a paper ballot, entering a prototype-issued ID and self-reporting age, gender, and ethnicity. In condition (B), participants used a gaze-only interface at the screen with no keyboard or mouse input; the system logged gaze coordinates to infer the selected candidate and used FR+ML to estimate age, gender, and ethnicity in real time. Consent was obtained (see Section 6.10).

Measures included the distribution of candidate choices (counts and percentages) and agreement between real (paper) and predicted (gaze-based). Statistical comparisons used 2×K$\chi^{2}$ tests, total-variation distance |Δ|, and per-class percentage point deltas.

Results (see Section 6.2, Section 6.3, Section 6.4 and Section 6.5) show close alignment between real and inferred distributions except for age. For candidate choice, the global gap is |Δ|=6.67% with $\chi^{2}\left(8\right)=2.28$, p=0.971 (Cramér’s V=0.12); ethnicity shows |Δ|=5.33% with $\chi^{2}\left(4\right)=6.01$, p=0.199; and gender shows |Δ|=2.7% with $\chi^{2}\left(1\right)=0.25$, p=0.621. These alignments should be interpreted in light of the highly structured task and short decision horizon, conditions under which visual attention is known to closely precede selection. In contrast, age exhibits a substantial distributional gap (|Δ|=40.0%, KS D=0.307, $\chi^{2}\left(3\right)=28.71$, $p\approx 2.6\times 10^{-6}$), reflecting a systematic overestimation in the 21–25 bin.

Accordingly, the present study should be viewed as a systems-oriented and methodological contribution to consumer research, providing an operational platform for multimodal analysis rather than a definitive model of consumer preference formation.

### 6.9. Post-Session Questionnaire: Performance and UX (2 October 2025)

**Figure 29:**
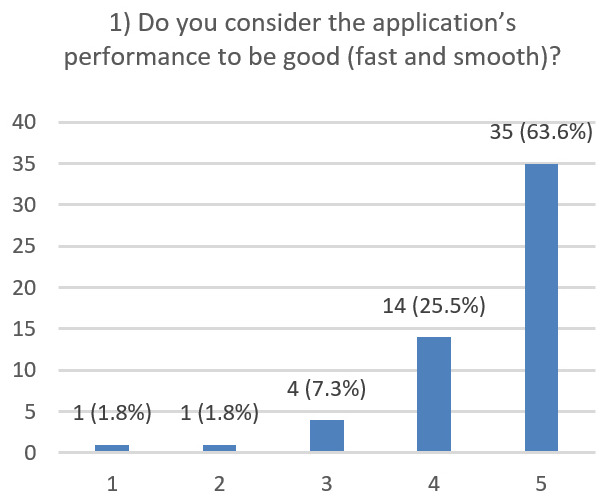
Q1: Application performance (Likert 1–5).

**Figure 30:**
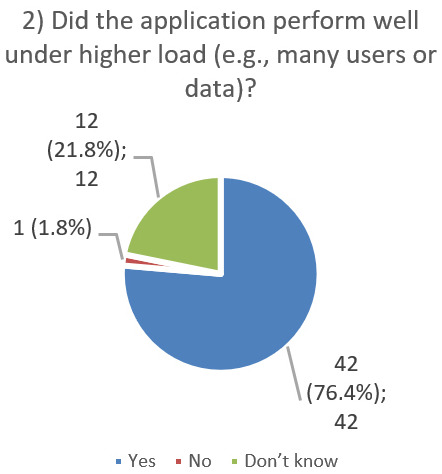
Q2: Performance under higher load (Yes/No/Don’t know).

**Figure 31:**
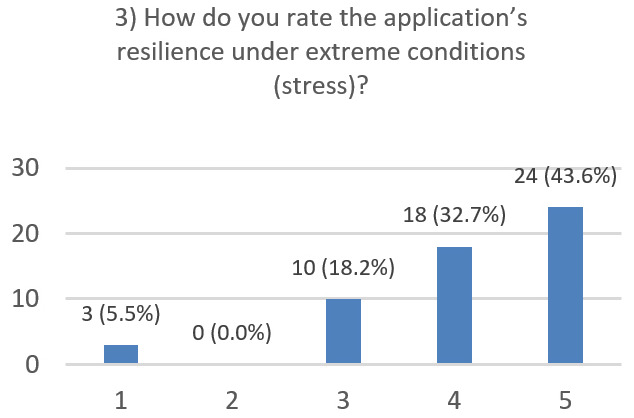
Q3: Resilience under stress (Likert 1–5).

**Figure 32:**
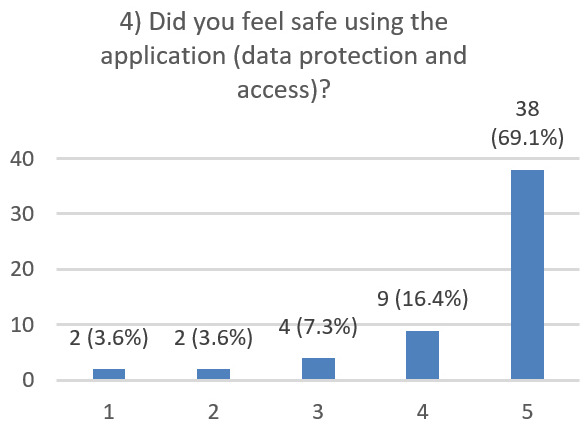
Q4: Perceived safety (Likert 1–5).

**Figure 33:**
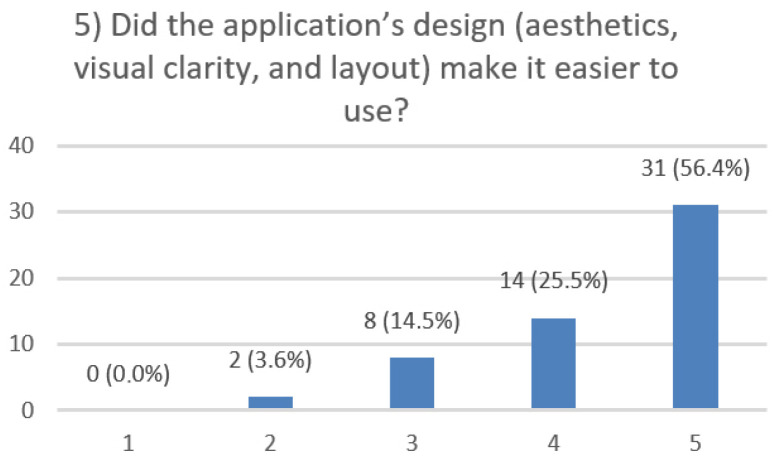
Q5: Design aided usage (Likert 1–5).

**Figure 34:**
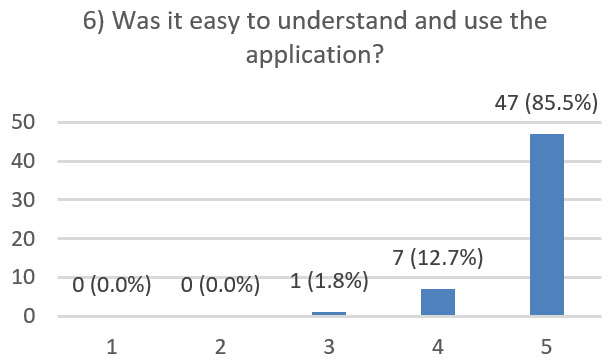
Q6: Ease of understanding and use (Likert 1–5).

**Figure 35:**
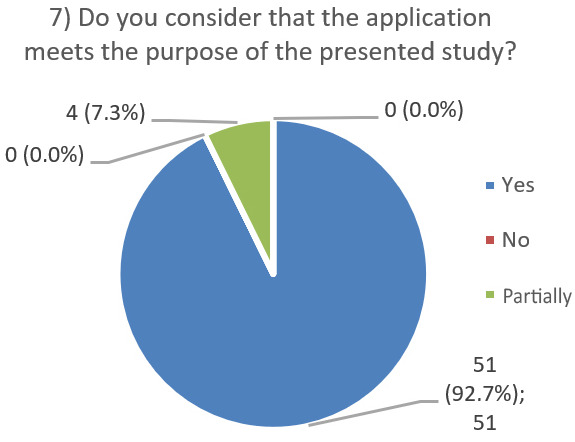
Q7: Meets study purpose (Yes/No/Partially/Don’t know).

**Figure 36:**
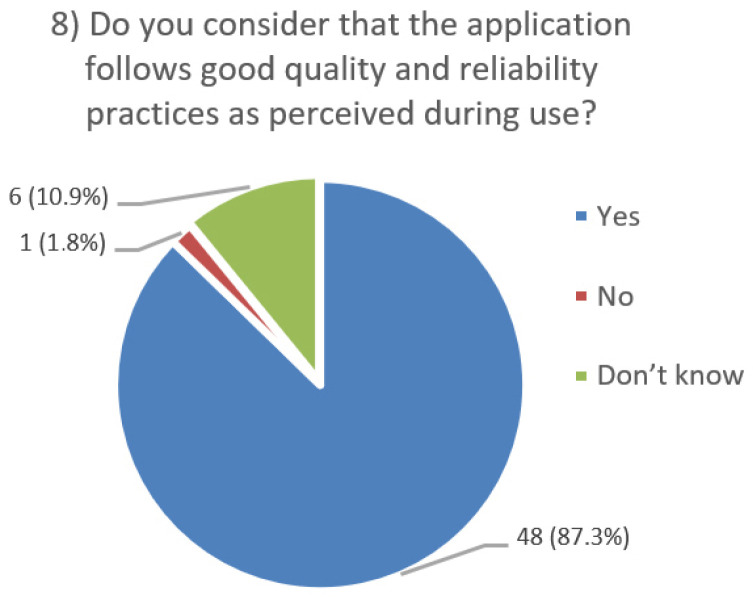
Q8: Quality and reliability (Yes/No/Don’t know).

**Figure 37:**
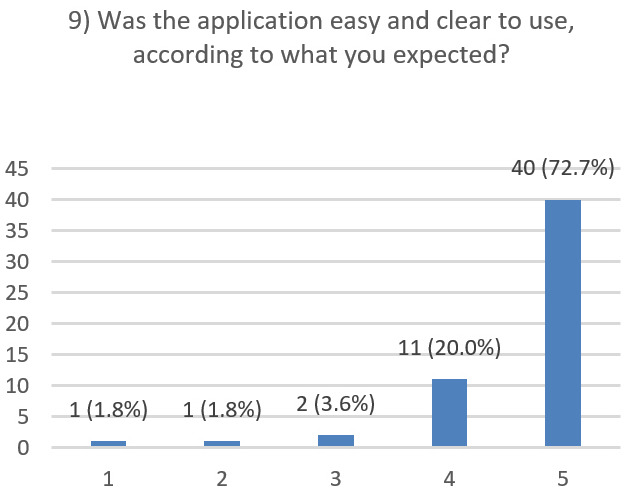
Q9: Clarity/ease versus expectations (Likert 1–5).

**Figure 38:**
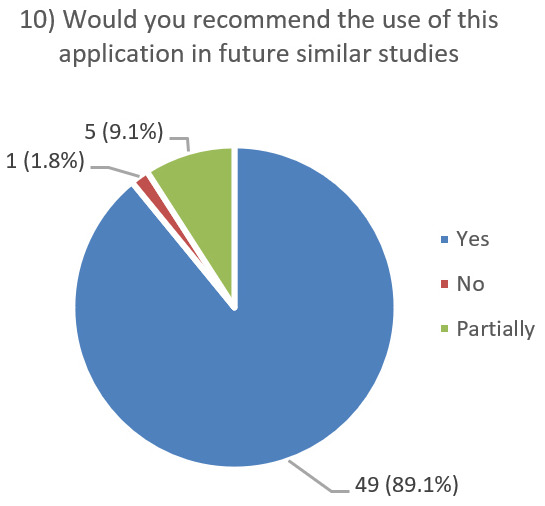
Q10: Recommendation for future studies (Yes/No/Partially).

After using the prototype on-screen (gaze-only condition), participants completed a 10-item Likert questionnaire (Figure 29, Figure 30, Figure 31, Figure 32, Figure 33, Figure 34, Figure 35, Figure 36, Figure 37 and Figure 38) covering the following: application performance (fast and smooth), performance under higher load, resilience under stress, perceived safety (data protection and access), design-aided usage (aesthetics, visual clarity, layout), ease of understanding and use, fit to the stated study purpose, perceived quality and reliability practices, clarity/ease versus expectations, and recommendation for future similar studies. The objective was to assess performance and UX in a realistic post-interaction setting.

Parallel records comprised the paper ballot (condition A) and logs of demographic predictions (FR/ML: age, gender, ethnicity) and the candidate selected via gaze, with timestamp, participant ID, and study ID.

Planned metrics included mean ± SD per item, an overall score (mean of the 10 items), Cronbach’s α, percentile distributions, cross-tabs by real vs. predicted demographics, Spearman correlation between the overall score, and real vs. pred agreement on candidate choice and outlier reporting.

### 6.10. Ethics, Privacy, and Data Protection

All participants provided informed consent prior to data collection and were informed about the modalities captured (gaze and transient webcam frames), the purpose of processing, and their right to withdraw. Data were collected and processed solely for the author’s PhD research, handled confidentially, and not shared with third parties for commercial use. Webcam frames are captured transiently and used exclusively to perform on-device facial analysis for demographic inference (age, gender, and ethnicity). Raw images are not retained: all webcam frames are automatically deleted immediately after inference. Only the resulting demographic predictions and their timestamps are stored, together with study/session identifiers that are not linked to real-world identities. No names, contact details, biometric templates for identity recognition, or other direct identifiers are collected at any stage. Gaze-derived outcomes (e.g., AOI metrics, dwell time, inferred choice) are computed from eye tracking samples and stored under pseudonymous study/session IDs. Demographic attributes are used exclusively as contextual metadata for anonymised, aggregate and segment-level analysis (e.g., comparing attention patterns across age/gender/ethnicity strata). They are not used as predictive features for choice inference and are not employed for individual identification, profiling, targeting, or decision-making about participants. Data handling follows GDPR principles of purpose limitation, data minimisation, and storage limitation. Access to stored records is restricted to authorised research personnel, and data are stored in secured local infrastructure with appropriate access controls. Where applicable, participants may request access, correction, or deletion of their data in accordance with GDPR.

#### Fairness, Misclassification Risk, and Usage Limits

Automatic demographic inference is inherently fallible and may be uneven across subgroups; in marketing and UX settings, misclassification can distort segment reports and, if misused, reinforce stereotypes or lead to exclusionary decisions. For this reason, demographic inference in DeepVisionAnalytics is optional and can be disabled at study configuration time; the core behavioural signal and choice inference remain gaze-driven. When enabled, demographics are reported only at aggregate level, using minimum-group reporting thresholds (k-anonymity-style suppression of small strata) to avoid over-interpretation and reduce privacy risk. We recommend routine bias monitoring and calibration per subgroup (e.g., subgroup error and confidence diagnostics for age bins, gender, and ethnicity classes) and explicit documentation of known failure modes (lighting, pose, camera domain shift). The system is not intended for sensitive targeting, eligibility decisions, or personalised persuasion; demographic attributes are used solely for research segmentation under human oversight and auditable reporting.

## 7. Discussion

This work set out to examine whether an integrated eye tracking (ET), computer vision (OpenCV), and multitask machine learning pipeline can recover reliable, low-latency behavioural signals of visual attention in a realistic interaction setting, and whether these signals can be leveraged to support the analysis of consumer choice under constrained conditions. Across tasks, the results indicate that objective behavioural streams—gaze traces and fixations—can be fused with automatic demographic estimates (age, gender, ethnicity) to approximate survey-style outcomes in structured tasks, while reducing reliance on self-reported measures.

Principal findings. First, the gaze-only classifier reproduced the paper ballot distribution of candidate choices with high accuracy (85.33%) and no statistically significant distributional difference ($\chi^{2}\left(8\right)=2.28$, p=0.971; |Δ|=6.67%), with strongest recall on classes with larger support (A/C/D) and most errors concentrated in B → A confusions. Second, the demographic heads showed mixed performance: gender and ethnicity closely matched the real distributions ($\chi^{2}$ non-significant; |Δ|=2.7% and 5.33%, respectively), whereas age exhibited a clear upward bias concentrated in the 16–20 bin (|Δ|=40.0%; KS D=0.307). Third, the prototype was judged usable and responsive (overall ≈4.3–4.4/5 on a 0–5 Likert scale), with the only consistent watchpoint being offline operation (B16).

Interpretation and implications. The close correspondence between gaze-based selections and paper ballots indicates that, in highly structured and visually driven tasks with short decision horizons, measured attention can approximate observed choice outcomes. This finding complements prior work emphasising that attention does not constitute a general proxy for preference; rather, its interpretive value is conditional on task structure, stimulus salience, and interaction constraints. In the present setting, the ET signal, paired with minimal interface friction, proved sufficient to elicit stable selections in real time. Practically, this supports replacing limited portions of self-report workflows with unobtrusive ET in scenarios such as package A/B tests, hero-image evaluations, or shelf-layout comparisons, where decisions are immediate and visually anchored. Residual class-specific errors (e.g., under-allocation to B) motivate light per-class calibration (threshold tuning or class-weighted losses) prior to operational deployment.

Role of demographic context. Importantly, the results summarised in Table 6 make explicit that candidate choice in the present study is inferred exclusively from gaze behaviour. Demographic attributes (age, gender, ethnicity) are not used as predictive features for choice inference and do not influence the decision process. Instead, they are linked post hoc to gaze-derived outcomes to enable stratified and segment-level interpretation of observed choices. This distinction is essential to avoid conflating behavioural prediction with demographic priors. The contribution of demographic inference in this framework is therefore interpretative rather than decisional: it supports contextual analysis and validation across segments without implying that demographic profiles determine individual preferences.

Age bias and model calibration. The upward shift observed in the age head—median increase of approximately one year, wider interquartile range, and heavier upper tail—is consistent with training data imbalance and domain shift (e.g., lighting, pose, and camera optics) between the training corpus and the study environment. Because age-sensitive segmentation and analysis can amplify bias, we recommend (i) age-dependent post hoc calibration (e.g., piece-wise-linear adjustment or quantile mapping), (ii) re-training with balanced, environment-matched samples (particularly in the 16–20 range), and (iii) periodic drift monitoring using small verified panels. These measures are expected to narrow dispersion and reduce KS/$\chi^{2}$ discrepancies without compromising latency.

Methodological contribution. Architecturally, the five-module design (Digital, Physical, Connection, Service, Data) proved effective for low-latency multimodal fusion. ET and FR/ML streams were synchronised on-device (<100 ms), enabling per-fixation demographic joins and interpretable, audit-ready outputs. Beyond descriptive visualisations, the system supports outcome-level evaluation through explicit performance metrics (recall, precision, confusion matrices, $\chi^{2}$, and |Δ| diagnostics) that can be directly interpreted by practitioners.

Alignment with the intended contribution. The empirical results support the four main contributions outlined in Section 3.1. First, the prototype operates as a reusable platform rather than a one-off experimental setup, having been instantiated across multiple study configurations. Second, real-time fusion of ET with automatic demographic inference demonstrates the feasibility of a multimodal feature space that can be reused for downstream analyses of choice, engagement, and task performance. Third, the real vs. pred validations for age, gender, ethnicity, and category choice show that the framework extends beyond heatmap-based reporting to calibrated predictive modelling. Finally, the generated outputs—AOI rankings, segment-level summaries, and confusion-based diagnostics—illustrate how behavioural signals can be translated into decision-support artefacts for marketing and UX teams.

Usability and adoption. High ratings across usability blocks and strong agreement on clarity, responsiveness, and ET↔FR synchronisation lower the barrier to adoption in laboratory and pilot retail settings. The main limitation—offline robustness—should be addressed through local caching, retry queues, and graceful degradation of FR/ML services when connectivity is constrained.

Threats to validity. Sample sizes (N = 75 for Real vs. Pred; N = 7 for the usability panel) limit statistical power for rare classes and restrict external validity. The use of salient facial stimuli further constrains generalisability to cluttered environments or long-horizon outcomes (e.g., recall or purchase). Moreover, automatic demographic inference introduces fairness risks if training data remain imbalanced; ongoing audits using per-class reliability curves and fairness metrics are therefore essential.

Ethics and governance. Even with consent and GDPR-aligned handling, demographic estimation introduces fairness risks in applied marketing contexts: misclassification may bias segmentation, reinforce stereotypes, or contribute to exclusion if used to steer offers or experiences. We therefore treat demographics as a non-essential, switchable layer: studies can rely purely on gaze/AOI metrics when demographic context is not necessary for the research question. When demographics are enabled, results should be interpreted only at the group level (with suppression of small strata) and accompanied by subgroup calibration/monitoring (error rates, reliability curves, and drift checks by subgroup), with human review of any high-impact conclusions and transparent documentation of limitations.

### Managerial Takeaways

For marketing and UX practitioners, the results of this study suggest the following practical implications:

- The platform can be integrated into existing research workflows as a lightweight screening layer prior to large-scale surveys or lab studies, supporting rapid iteration in early design and concept testing phases.
- Eye tracking can replace or complement short self-report choice tasks in visually driven evaluations (e.g., package A/B tests, hero images, shelf layouts) where decisions are immediate and time-constrained and option sets are limited.
- Gaze-based outcome inference is most reliable for low-friction, goal-directed tasks; it should not be used as a proxy for preference in exploratory, emotional, or identity-driven decision contexts.
- Compared to traditional lab-based neuromarketing studies and repeated survey instruments, the proposed setup reduces per-study cost and participant burden, while preserving objective behavioural measurement and auditability.
- Demographic attributes should be used exclusively for post hoc segmentation and contextual interpretation, not as inputs to automated decision-making or individual-level targeting.
- The system is best positioned as a decision-support tool for comparative evaluation (e.g., ranking alternatives, identifying attention bottlenecks), rather than as a standalone predictor of long-term attitudes or market outcomes.

Future directions. Future work will prioritise field deployments, integration of complementary physiological signals, and adaptive learning. Additional priorities include active learning to close class gaps (e.g., age 16–20), domain adaptation to improve robustness under variable capture conditions, and human-in-the-loop review of high-uncertainty cases. Overall, the results indicate that real-time ET combined with lightweight FR/ML can deliver decision-grade, context-aware insights with reduced participant burden. With targeted calibration, subgroup-level fairness monitoring, k-anonymity-style aggregate reporting, and offline hardening, the framework is well positioned for larger-scale validation in marketing research and adjacent UX contexts.

## 8. Limitations and Future Work

Table 7 summarises the main strengths of the proposed framework alongside its current limitations, providing context for the discussion that follows.

Limitations. First, ecological validity is constrained by the controlled environment and specific task (candidate choice using prominent faces), which may not fully reflect real-world interactions such as cluttered shelves, complex interfaces, or longitudinal exposure. Second, the modest sample sizes (N = 75 for Real vs. Pred; N = 7 for usability) limit the precision of estimates for rare classes and reduce power for detecting small effects. Third, even with informed consent and GDPR-compliant procedures, any system based on ET and automatic facial analysis raises ongoing privacy, ethics, and fairness concerns that must be monitored as the prototype moves towards operational use. Effect sizes (e.g., total-variation distance |Δ| and Cramér’s *V*) are reported throughout to contextualise statistical significance and to support cautious interpretation given the exploratory, proof-of-concept nature of the study.

Future work. Future work should address these limitations along three main lines. First, larger-scale deployment in retail and online settings is needed to validate performance under more heterogeneous conditions and for different task types (e.g., packaging, advertising, UX flows). Second, integrating additional physiological sensors (e.g., heart rate, EDA) could provide richer affective context and help disentangle attention, arousal and preference. Third, adaptive and online learning methods should be explored to support real-time model refinement, including active learning for under-represented classes, domain adaptation to new environments, and systematic calibration procedures for demographic and choice predictions.

## 9. Conclusions

This paper introducedDeepVisionAnalytics, a reusable platform for consumer behaviour analysis that integrates eye tracking, OpenCV-based computer vision, and machine learning-based facial analysis within a single end-to-end workflow. In the present study, candidate choice was inferred exclusively from AOI-level gaze behaviour in a constrained, visually driven task, while demographic estimates (age, gender, ethnicity) were obtained independently and linked post hoc to support stratified, segment-level interpretation rather than to influence the choice inference process.

Empirically, the gaze-only classifier closely reproduced paper ballot choices (85.33% accuracy), and distributional agreement was high (|Δ|=6.67%; $\chi^{2}$ non-significant). Demographic validation showed strong alignment for gender and ethnicity, whereas age exhibited an upward bias that motivates calibration before using age-based segmentation. Methodologically, the five-module architecture demonstrates that it is feasible to move beyond descriptive heatmaps by producing audit-ready behavioural outputs (AOI metrics, rankings, and confusion-based diagnostics) together with segment-specific summaries derived from post hoc demographic linking. Practically, the prototype delivers reusable decision-support artefacts (e.g., heatmaps, AOI rankings, and segment-level reports) that can inform product and interface optimisation and the design of future studies.

Future work should extend validation to broader tasks and populations, harden calibration and fairness monitoring for demographic inference, and integrate additional signals only where they add explanatory value. With these developments, DeepVisionAnalytics can support more precise, data-driven marketing and UX analysis while maintaining clear separation between gaze-driven outcome inference and demographic contextual interpretation.

## Figures and Tables

**Table 1:** The selected articles’ research topics.

Topic	References
UX/UI	[12,13,14,15,16,17,18,19,20,21,22]
Advertising/digital persuasion	[23,24,25,26,27]
Packaging/shopper research	[1,2,5,28,29,30,31,32,33,34,35,36]

**Table 2:** Comparison between DeepVisionAnalytics and commercial research suites.

Capability	DeepVisionAnalytics	Commercial Suites (e.g., iMotions)
Analytical role	Gaze infers choice; demographics are linked post hoc to contextualise interpretation.	Primarily descriptive gaze analysis with auxiliary measures.
Multimodal integration	Native real-time ET + facial analysis with explicit separation between inference and context.	ET combined with surveys/biosignals; facial analysis often optional or offline.
Outcome modelling	Formal gaze-based outcome inference with quantitative validation.	Heatmaps and gaze metrics; outcome inference typically external or manual.
Transparency and control	Audit-ready raw logs (SQL + JSON) and full control over models and analytics.	Session-level exports with limited access to raw, time-aligned data.
Deployment model	Fully local, offline-capable research platform.	Licensed software stacks with limited offline support.

**Table 3:** Key contributions of DeepVisionAnalytics compared with prior eye tracking studies in marketing and UX.

Dimension	Prior Studies (Typical)	DeepVisionAnalytics (This Work)
Research scope	Single-study, single-stimulus experiments tailored to a specific product, interface, or campaign.	Reusable, multi-study platform supporting heterogeneous marketing and UX scenarios under a unified pipeline.
Role of eye tracking	Primarily descriptive diagnostics (heatmaps, dwell time, scan paths).	Behavioural signal used for explicit outcome inference under constrained tasks, with quantitative validation.
Use of demographics	Self-reported or absent; occasionally used as grouping variables.	Automatically inferred and linked *post hoc* as contextual metadata, explicitly separated from the decision mechanism.
Multimodal integration	Limited or offline fusion of gaze with other measures (surveys, physiology).	Real-time, on-device integration of eye tracking and facial analysis with time-aligned logs.
Analytical outputs	Study-specific visualisations and statistical tests.	Audit-ready artefacts: AOI rankings, inferred choices, segment-level summaries, and confusion-based diagnostics.
Reusability and extensibility	Bespoke setups with limited transferability across studies.	Configurable architecture designed for replication, extension, and cumulative learning across contexts.

**Table 4:** Age distribution alignment before and after post hoc bin-wise bias correction (N=75).

Metric	Before	After	Change
Max bin deviation $\max_{b}\left|\Delta_{b}\right|$ (pp)	40.0	57.3	+17.3
KS statistic *D*	0.307	0.573	+0.266
MAE (years)	3.35	4.60	+1.25

**Table 5:** Confusion matrix (Real × Pred) for candidate choice inferred from gaze (N=75).

Real\Pred	Cand A	Cand B	Cand C	Cand D	Cand E	Cand F	Cand G	Cand H	Don’t Know	Total
Cand A	20	0	0	0	0	0	2	0	0	22
Cand B	3	13	0	1	0	0	0	0	0	17
Cand C	1	0	15	0	0	0	0	0	0	16
Cand D	0	0	0	10	0	1	0	0	0	11
Cand E	0	1	0	0	2	0	0	0	0	3
Cand F	0	0	0	0	1	1	0	0	0	2
Cand G	0	0	0	0	0	0	2	0	0	2
Cand H	0	0	0	0	0	0	0	1	0	1
Don’t know	0	0	0	0	0	1	0	0	0	1
Total Pred	24	14	15	11	3	3	4	1	0	75

**Table 6:** System-level comparison showing how demographic context is used only for post hoc, segment-level interpretation of gaze-inferred candidate choice (N=75).

Model/Analysis	Feature Set	Accuracy (%)	Macro-F1 (%)	|Δ| (%)
Gaze-only choice inference	AOI-level gaze metrics (e.g., dwell time, time-to-first-fixation, revisits, fixation count), aggregated per stimulus and choice window.	85.33	70.20	6.67
Demographic context (descriptive)	Predicted age, gender, and ethnicity obtained from independently trained CNN models (pre-trained .h5) and linked *post hoc* to gaze-derived outcomes for stratified analysis; not used as predictive inputs for choice inference.	N/A	N/A	N/A

*Note:* N/A denotes non-applicable metrics, since demographic variables are used exclusively for post hoc descriptive analysis and not for prediction.

**Table 7:** Strengths and limitations of the proposed framework.

Strengths	Limitations
Objective behavioural measurement based on gaze rather than self-report.	Evaluation conducted in controlled, short-horizon tasks with limited ecological complexity.
Real-time, low-latency multimodal fusion with audit-ready logs.	Automatic demographic inference is sensitive to training imbalance and domain shift.
Reusable platform supporting multiple marketing and UX scenarios.	Results should be interpreted as proof-of-concept rather than generalisable evidence.
Transparent validation using distributional metrics and effect sizes.	Long-term outcomes (e.g., recall, purchase behaviour) are not captured.

## Data Availability

Data are contained within the article.
